# Multilevel Direct Repair Surgery for Three-Level Lumbar Spondylolysis

**DOI:** 10.1155/2013/472968

**Published:** 2013-03-28

**Authors:** Tetsu Arai, Koichi Sairyo, Isao Shibuya, Ko Kato, Akira Dezawa

**Affiliations:** Department of Orthopedic Surgery, Teikyo University Mizonokuchi Hospital, Kawasaki 213-8507, Japan

## Abstract

A 45-year-old man presented to our clinic requesting evaluation for surgical treatment of chronic low back pain of more than 20 years duration. He was diagnosed with 3-level lumbar spondylolysis at L3–5. Direct repair using the pedicle screw and hook-rod system was conducted for all three levels. After the surgery, his low back pain completely disappeared. Six months later, he felt discomfort and heard a metallic sound as he twisted his trunk. Computed tomography and radiography indicated that the hook head for L3 and the screw head for L4 were interfering with each other, causing the sound. We confirmed bony union at L3 and removed the L3 system. Surgeons should be aware of such complications if direct repair using a pedicle screw and hook-rod system is conducted for multilevel spondylolysis.

## 1. Introduction

Lumbar spondylolysis is a stress fracture of the pars interarticularis and usually occurs in children and adolescents [1–3]. Sakai et al. reviewed 2,000 computed tomography (CT) scans taken for abdominal disease, such as gastrointestinal and gynecological disease [4], and found 117 cases of lumbar spondylolysis among them, giving a prevalence of 5.9% for spondylolysis in the Japanese general population. Only 5 of the 117 cases were multilevel spondylolysis: 3 cases were 2-level disease (0.2%) and 2 cases were 3-level disease (0.1%).

Lumbar spondylolysis is a benign clinical condition, and in the adult population it is reported that this disorder is unlikely to cause backache [5–7]. When it does cause back pain and cannot be controlled with conservative treatment, surgical intervention is indicated. For painful lumbar spondylolysis, direct repair surgery is suitable in case without spondylolisthesis [8–12]. Recently, a minimally invasive technique of direct repair has been reported [13, 14]. However, there are very few reports of direct repair surgery for multilevel lumbar spondylolysis due to its rarity.

In this paper, we discuss the clinical problems associated with multilevel direct repair for 3-level spondylolysis (L3–5).

## 2. Case Presentation

A 45-year-old man presented to our clinic requesting evaluation for surgical treatment of chronic low back pain of more than 20 years duration. He started Judo at the age of 12 and suffered severe low back pain at age 17 and could not practice Judo for 2 months due to the pain. He did not visit a hospital that time and did not receive a diagnosis for this pain. It disappeared within 2 months of stopping Judo practice and resting at home. After this episode, he started to suffer from chronic low back pain. He continued Judo until he graduated from technical college and became a sailor.

During his first year at sea, he experienced very strong back pain again and visited a hospital for the first time. Triple-level spondylolysis at L3–5 was diagnosed from plain radiographs. The pain subsided with a NSAID (nonsteroidal anti-inflammatory drug) and sick leave. For more than 20 years after, he suffered from strong back pain, requiring sick leave for a couple of days, 3 to 4 times a year. He consulted many orthopedic surgeons to solve the problem, and all doctors recommended NSAIDs and rehabilitation. He visited us seeking possible surgical treatment.

**Figure 1:**
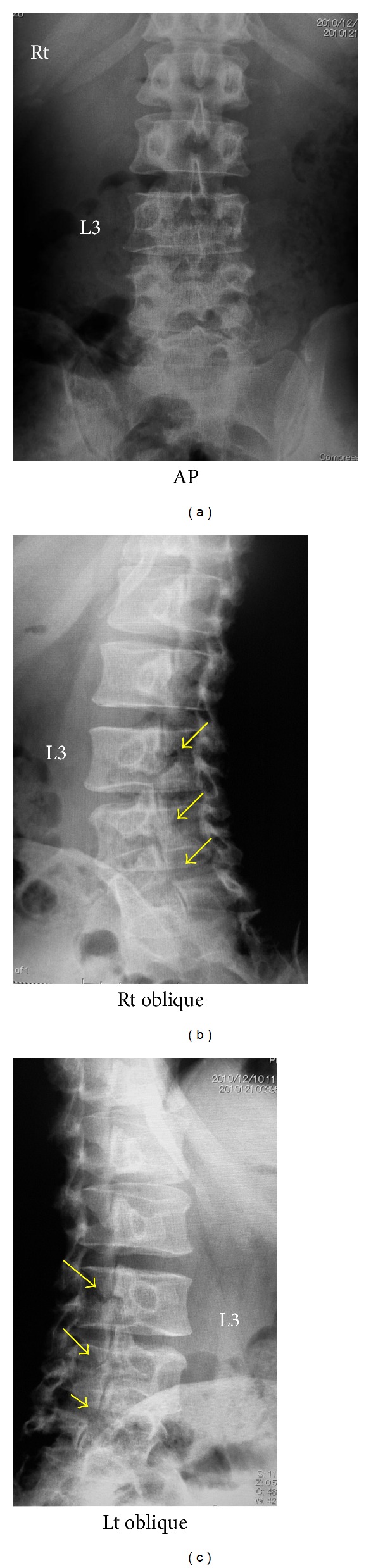
Plain anteroposterior and oblique radiographs at initial presentation show bilateral lumbar spondylolysis at L3–5.

**Figure 2:**
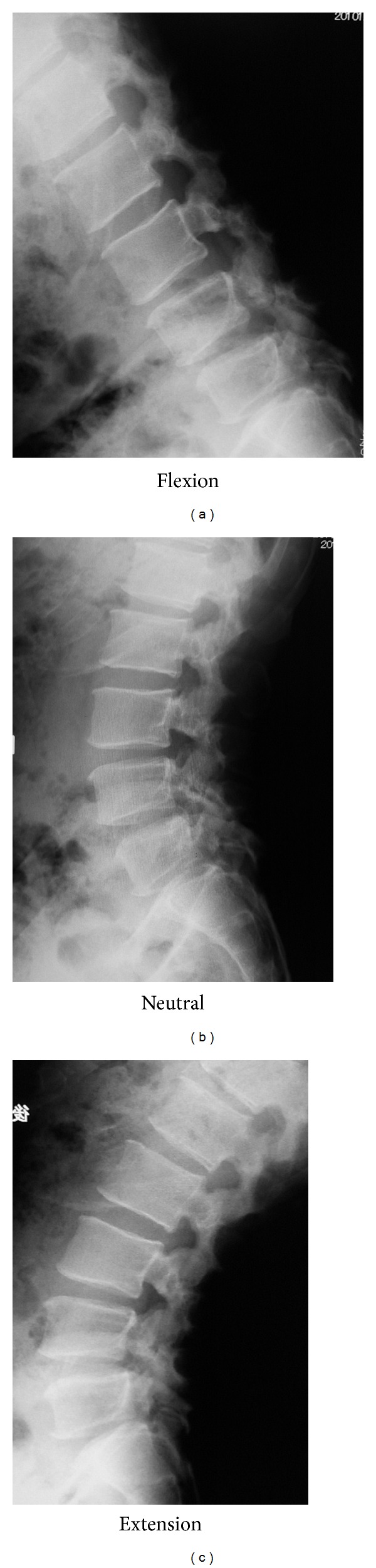
Plain dynamic film at initial presentation shows no obvious instability.

**Figure 3:**
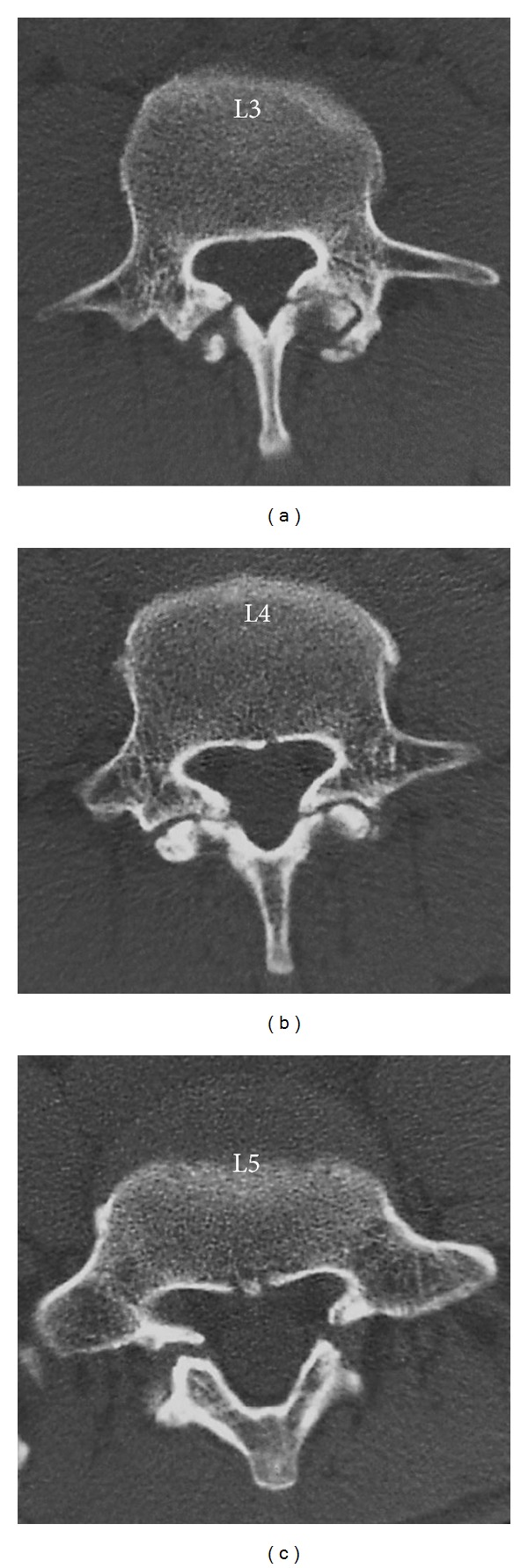
CT at initial presentation shows pseudarthrosis-type pars defects bilaterally at L3–5.

**Figure 4:**
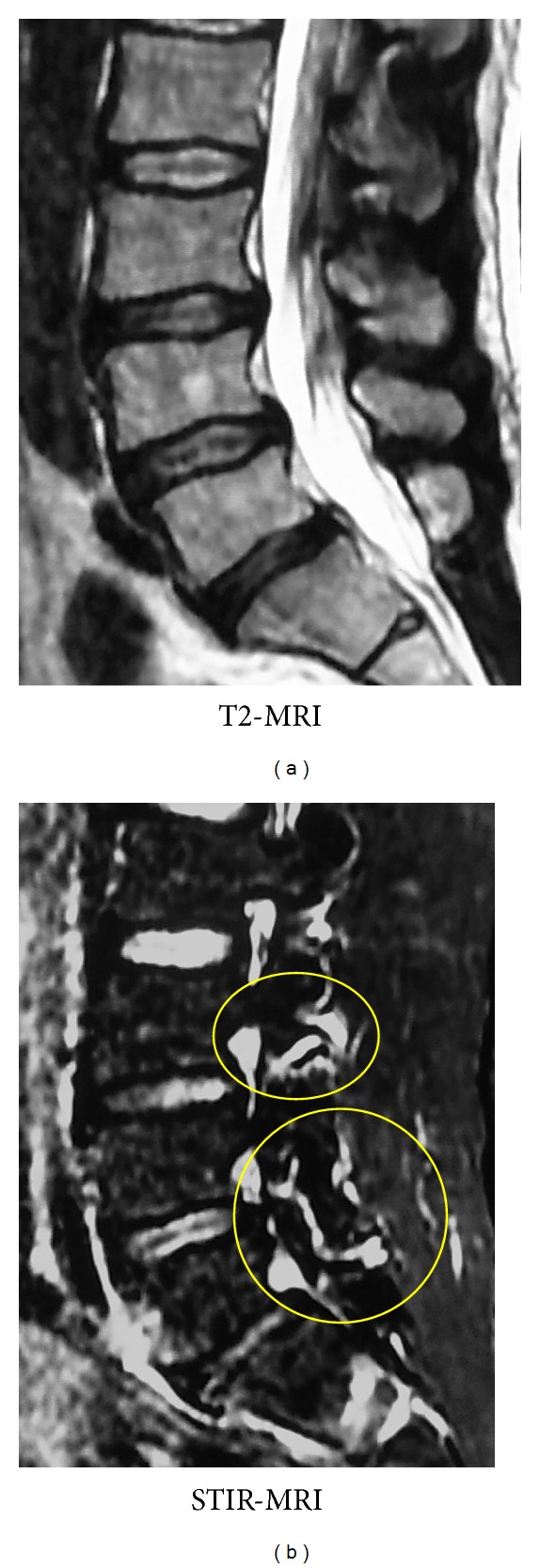
MRI at initial presentation shows in the left panel no obvious degenerative spinal disorder such as herniated nucleus pulposus or spinal canal stenosis. Slight disc degeneration at L3-L4, L4-L5, and L5-S1 is evident. In the right panel, effusion is also seen around the pars defects and surrounding facet joint.

Plain radiographs from his first visit to our clinic are shown in Figure 1. Bilateral spondylolysis at L3–5 is evident on oblique films. There is no instability apparent on dynamic films (Figure 2). CT scan (Figure 3) demonstrated pseudarthrosis at all three sites of spondylolysis, which cannot be expected to achieve bony healing with conservative care [15]. On magnetic resonance imaging (MRI) (Figure 4(a)), no other degenerative spinal disorders such as herniated nucleus pulposus or spinal canal stenosis were found. Slight disc degeneration at L3-4, L4-5, and L5-S1 was seen. Effusion was also seen around the pars defects and surrounding facet joints (Figure 4(b)), indicating inflammation in the space [16].

On initial presentation, he rated his low back pain at 1-2 out of 10 on a visual analog scale. The pain increased on lumbar extension but not flexion. Tenderness was noted on the spinous processes of L3–5. All neurological findings were normal, and there were no positive tension signs, including the femoral nerve stretch test and the straight leg raise test. It was difficult to decide the surgical indication for this case, since usually his pain was moderate at consultation. Usually, direct repair surgery would be indicated after confirming the pain to originate from the defects by steroid infiltration into them [13, 14]. In this case, we decided to conduct direct repair surgery for all three levels since there were no other obvious degenerative disorders causing low back pain on radiological investigation.

**Figure 5:**
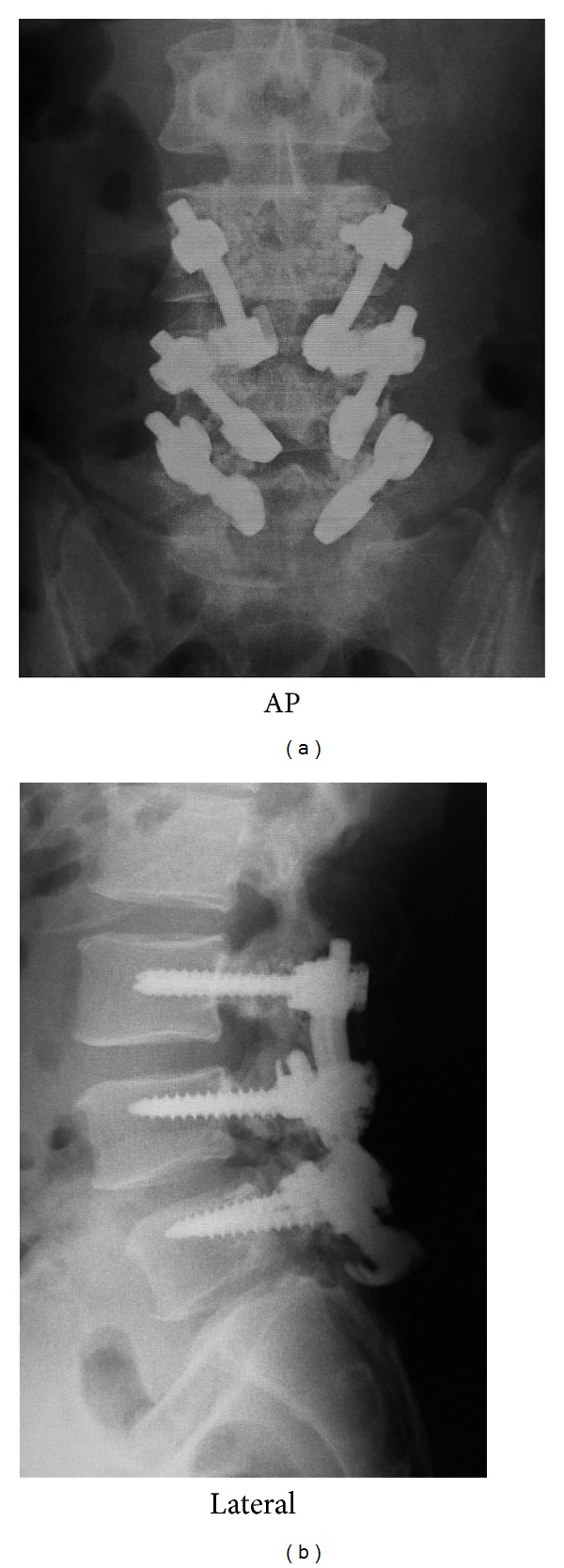
Three-level pars direct repair surgery. Note the pedicle screw and hook-rod system are used for each level.

**Figure 6:**
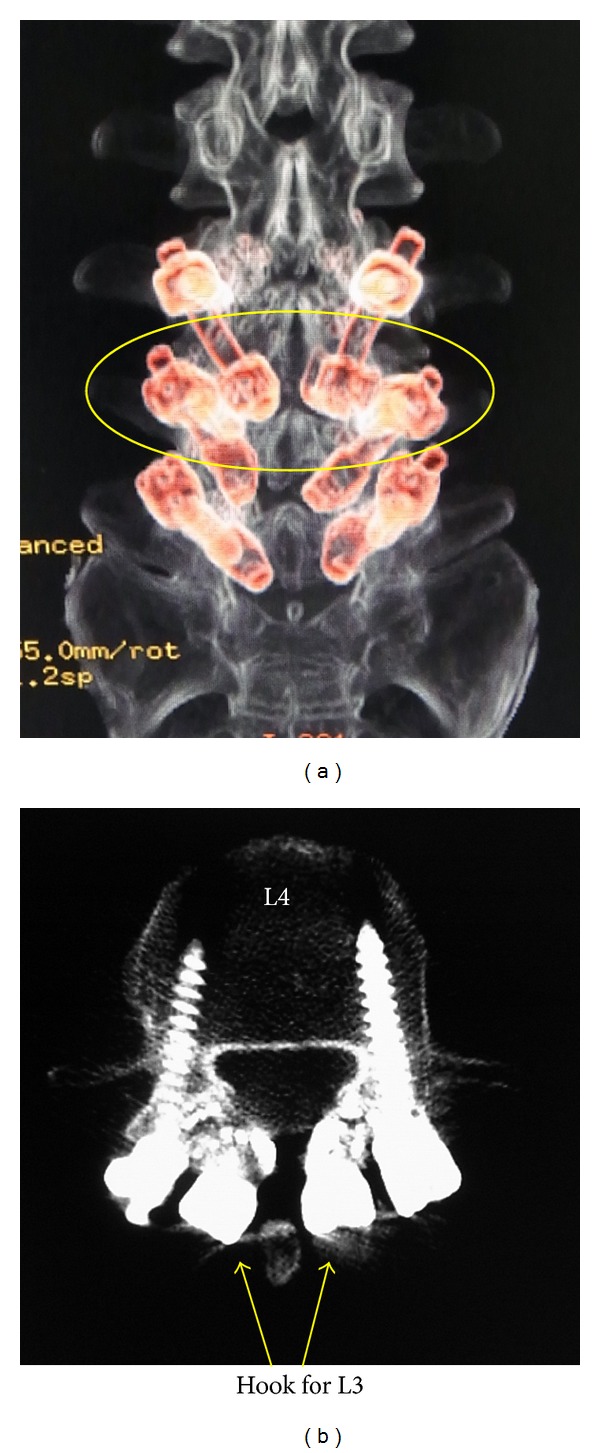
CT after the surgery. Note the proximity of the hook head to L3 and the pedicle screw head to L4.

**Figure 7:**
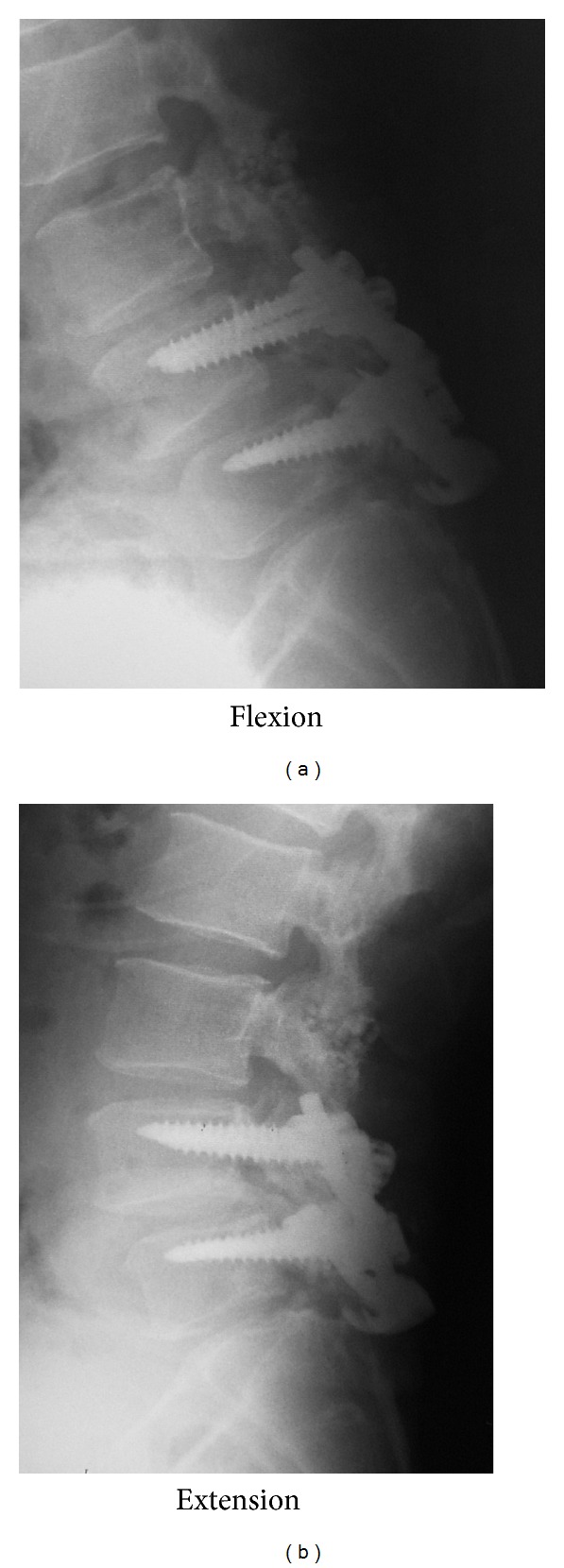
Plain dynamic film after the removal of the L3 systems. Motion is preserved.

A pedicle screw and hook-rod systems were utilized for L3–5. After debridement and decortication of the pseudarthroses, these systems were installed (Figure 5). Autologous bone was harvested from the iliac crest and was grafted on the defects. One month later, he returned to his job as a captain of an international passenger ship. His chronic back pain disappeared after the surgery. Six months after the surgery, he complained of an abnormal metallic sound and discomfort in his back during lumbar twisting motion. Amongst the implants, the hook for L3 and the pedicle screw head for L4 were closely located (Figure 6), and therefore we hypothesized that contact of these two components was causing the sound. After confirming bony union of the L3 pars defects, the L3 implants were removed. After removal, the noise and discomfort were resolved. At the 2-year followup, the patient had no complaints of low back pain and had not experienced any further pain attacks. Dynamic radiographs that demonstrated motion had been preserved (Figure 7).

## 3. Discussion

In this paper, we presented a patient that underwent 3-level direct repair surgery for lumbar spondylolysis at L3–5. Since the prevalence of 3-level spondylolysis is rare at 0.1% in the general population, it is very important that case reports of multilevel direct repair surgery be reported.

There are many types of direct repair surgery, including Scott's wiring [8], Buck's screwing [9, 13], the pedicle screw and hook-rod system [10, 11, 14], and the V-rod system [12]. Amongst these, we favor the pedicle screw and hook rod-system because pedicle screwing is a very familiar technique. In the current case, we found a pitfall of this pedicle screw and hook-rod system, however. When the system is utilized for multilevel repair, the hook head for the cranial level and screw head for the caudal level may interfere with each other. In our patient, the hook head for L3 and the pedicle screw head for L4 contacted each other in lumbar rotation, causing a metallic sound and discomfort in the back. The width of the lamina is small at the upper lumbar level, so to avoid this problem we must explore some additional techniques to install this system.

**Figure 8:**
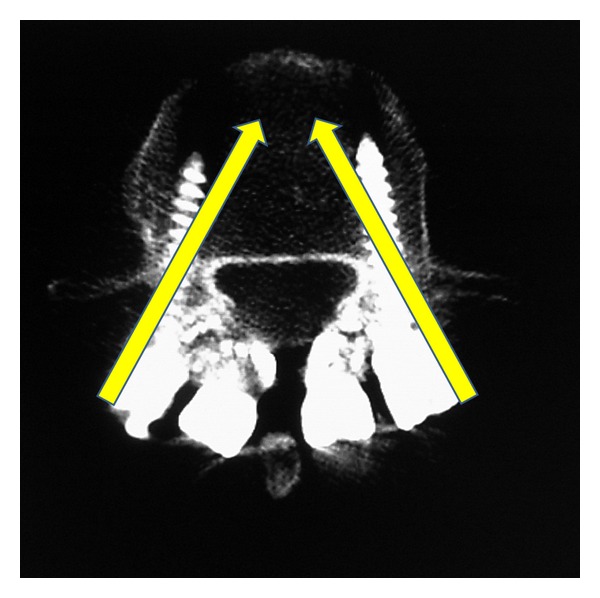
Optimum screw insertion point and direction for multilevel direct repair surgery for the pedicle screw and hook-rod system. The technique may avoid contact between the hook and screw heads.

Two possible solutions present themselves. First, the position of installation of hook or pedicle screw could be changed. However, as it is very difficult to change the position for hook, the insertion point of the pedicle screw could be changed instead. In our patient, as shown in Figure 6, pedicle screws were inserted near the facet joint. Therefore, to avoid contact with the hook head, the pedicle screw should be inserted from a more lateral position such as the base of the transversus process (Figure 8). The second alternative is to not use the hook system. Several techniques are available for direct repair surgery without using the hook system, including Scott's wiring [8], Buck's screwing [9, 13], and the V-rod method [12]. Based on a biomechanical investigation of these techniques, Scott's wiring would be weak [17], whereas Buck's screwing and the V-rod method showed similar biomechanical stability to the pedicle screw and hook-rod system [17]. For multilevel direct repair surgery, one of these two techniques would be preferable to avoid the problem we experienced in the current case.

In conclusion, we reported a case of 3-level direct repair for a patient with multilevel lumbar spondylolysis at L3–5. Clinically, his chronic back pain disappeared completely and he returned to work after the surgery. Complete resolution was complicated by contact between the hook head for L3 and the pedicle screw head for L4 in lumbar rotation, causing a metallic sound and discomfort in the back. For multilevel direct repair surgery, surgeons should be cognizant of this technical pitfall when using the pedicle screw and hook-rod system.
